# Enhancing the Sensing Performance of Zigzag Graphene Nanoribbon to Detect NO, NO_2_, and NH_3_ Gases

**DOI:** 10.3390/s20143932

**Published:** 2020-07-15

**Authors:** Ehab Salih, Ahmad I. Ayesh

**Affiliations:** 1 Department of Mathematics, Statistics and Physics, Qatar University, P.O. Box 2713, Doha, Qatar; e.salih@qu.edu.qa; 2 Center for Sustainable Development, Qatar University, P.O. Box 2713, Doha, Qatar

**Keywords:** zigzag graphene nanoribbon, adsorption energy, gas sensor, DOS

## Abstract

In this article, a zigzag graphene nanoribbon (ZGNR)-based sensor was built utilizing the Atomistic ToolKit Virtual NanoLab (ATK-VNL), and used to detect nitric oxide (NO), nitrogen dioxide (NO_2_), and ammonia (NH_3_). The successful adsorption of these gases on the surface of the ZGNR was investigated using adsorption energy (E_ads_), adsorption distance (D), charge transfer (∆Q), density of states (DOS), and band structure. Among the three gases, the ZGNR showed the highest adsorption energy for NO with −0.273 eV, the smallest adsorption distance with 2.88 Å, and the highest charge transfer with −0.104 e. Moreover, the DOS results reflected a significant increase of the density at the Fermi level due to the improvement of ZGNR conductivity as a result of gas adsorption. The surface of ZGNR was then modified with an epoxy group (-O-) once, then with a hydroxyl group (-OH), and finally with both (-O-) and (-OH) groups in order to improve the adsorption capacity of ZGNR. The adsorption parameters of ZGNR were improved significantly after the modification. The highest adsorption energy was found for the case of ZGNR-O-OH-NO_2_ with −0.953 eV, while the highest charge transfer was found for the case of ZGNR-OH-NO with −0.146 e. Consequently, ZGNR-OH and ZGNR-O-OH can be considered as promising gas sensors for NO and NO_2_, respectively.

## 1. Introduction

Graphene-based nanomaterials (G-NMs) have been subjected to intensive investigations in recent years due to their remarkable properties and promising application for electronics [1,2,3,4]. Nevertheless, it has been reported that graphene has some limitations in its sensing properties to some kinds of gas molecules such as CO, CO_2_, CH_4_, N_2_, NO_2_, NH_3_, and H_2_, affecting its usage for applications in the field of gas sensors [5,6,7,8]. These limitations can be solved either by functionalizing the surface of graphene or generating a G-NM which exhibits a tunable band gap that is referred to as a graphene nanoribbon (GNR) [9,10]. GNRs can be produced either by etching or patterning graphene along a specific direction [11,12,13]. In addition, practical challenges are still to be resolved in order to utilize graphene for practical sensors, such as its purity degree, trace metal content, application tests, and practical deposition of graphene-based materials on surfaces [14]. GNRs are considered a promising candidate for the field of gas sensors as compared with other C-NMs, thanks to their long and reactive edges that facilitate the adsorption of gas molecules [15,16,17,18,19,20]. Furthermore, GNRs have been reported in different forms of devices for utilization as a gas sensor, including field-effect transistors (FETs), chemiresistors, and capacitance sensors, etc. [21,22].

Although there have been great benefits to humans as a result of the continuous developments in technology and industry, there have been serious effects on human health due to the release of hazardous radiation [23,24,25] and substances such as toxic gases [26,27] and volatile organic compounds [28,29]. The presence of some of these gases, even at low concentrations, is very dangerous to human life [30]. Monitoring the level of nitrogen-based gases such as NO, NO_2_, and NH_3_ is of great interest for industry as well as medical applications. For instance, detecting NO molecules in the exhalation process can aid in the diagnosis of some respiratory diseases such as asthma [31,32]. Moreover, one of the main serious effects of NO gas is its easy transformation to the highly toxic NO_2_ gas through oxidation [33,34,35,36]. NO_2_ is a toxic gas which can be produced from diesel engines and combustion of fuel in industry [37]. Consequently, NO_2_-based gas sensors are of great importance to humans due to the high toxicity of NO_2_. These sensors have been used mainly in detecting pollutants in atmospheric air as well as the detection of explosive vapors [38,39]. On the other hand, monitoring and detecting NH_3_ gas molecules is a must in semiconductor and optoelectronic applications as well as for environmental purposes [40,41]. It has been found that annihilation of NH_3_ gas molecules at the 50–100 ppm level affects human health negatively through irritation of the throat, nose, and eyes [41,42]. Consequently, monitoring and detecting NO, NO_2_, and NH_3_ gas molecules are of great importance for overcoming some serious human problems such as air pollution. 

Therefore, the main concern of the current study is to improve the adsorption capacity of the zigzag graphene nanoribbon (ZGNR) towards NO, NO_2_, and NH_3_ gases by functionalizing its surface with epoxy and hydroxyl functional groups (-O- and -OH) to facilitate the adsorption of the gas molecules. Density functional theory (DFT), with the aid of the Atomistic ToolKit Virtual NanoLab (ATK-VNL), has been used to build bare ZGNR and to investigate its capacity to adsorb NO, NO_2_, and NH_3_ gases. The results reflected good adsorption parameters, indicating the ability of pure ZGNR to detect the three gases. To achieve the goal of this study, three new functionalized ZGNR systems were built: ZGNR-O, ZGNR-OH, and ZGNR-O-OH. The results showed a significant improvement in the adsorption energy, adsorption distance, and charge transfer between the gases and the three modified systems upon surface functionalization.

## 2. Computational Method

Four different ZGNR systems (bare ZGNR, ZGNR-O, ZGNR-OH, and ZGNR-O-OH) have been investigated by DFT calculations based on the ATK-VNL package in this study. Herein, DFT was utilized to relax and to optimize all atoms and systems until the convergence of force was 0.01 eV/Å. The resulting systems were then used as gas sensors to detect NO, NO_2_, and NH_3_ gases. The calculations were performed based on the generalized gradient approximation (GGA) of the Perdew–Burke–Ernzerhof (PBE) functional [43,44]. The density mesh cutoff was taken to be 125 Hartree and the force tolerance to be 0.01 eV/Å. Monkhorst–Pack k point sampling of 4 × 2 × 1 was used during all calculations. The successful adsorption of NO, NO_2_, NH_3_ gases on the surface of ZGNR systems was explored based on the adsorption energy ($E_{ads}$), adsorption distance (D), charge transfer (∆Q), band structure, and density of states (DOS). The adsorption energy of the gas molecules on ZGNR systems was calculated using the following formula [45,46,47]:

(1) $$E_{ads}=E_{ZGNR+gas}-(E_{ZGNR}+E_{gas})$$

where $E_{ZGNR+gas}$ is the total energy of the optimized structure of any gas molecules adsorbed on any of the different ZGNR systems. $E_{ZGNR}$ is the total energy of each of the ZGNR systems without gas adsorption, and $E_{gas}$ is the total energy of each of the optimized NO, NO_2_, and NH_3_ gases. Increasing the value of $E_{ads}$ with a negative sign indicates that the adsorption of NO, NO_2_, and NH_3_ gases on the surface of ZGNR systems is stronger [26,48]. Moreover, the charge transfer between the NO, NO_2_, and NH_3_ gases and the different ZGNR systems was calculated using Mulliken population analysis [49,50].

## 3. Results

### 3.1. ZGNR System

**Figure 1:**
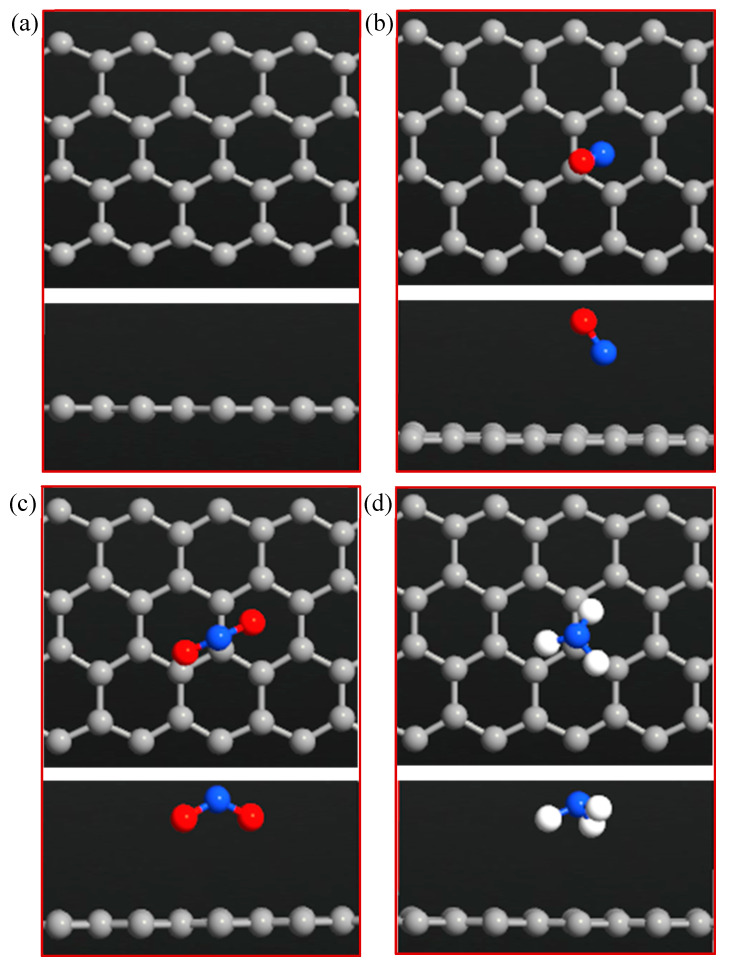
Top and side views of the optimized (**a**) ZGNR, (**b**) ZGNR-NO, (**c**) ZGNR-NO_2_, and (**d**) ZGNR-NH_3_.

The bare ZGNR system was prepared based on ATK-VNL and investigated as a gas sensor to detect NO, NO_2_, and NH_3_ gases. The optimized structures of ZGNR before and after adsorption of the three gases are given in Figure 1. The results show that the average C-C bond length of ZGNR is 1.425 Å. The adsorption of NO, NO_2_, and NH_3_ gases on the surface of ZGNR was explored based on the adsorption energy, adsorption distance, charge transfer, band structure, and DOS. The calculations show that the adsorption energy of NO on ZGNR was −0.273 eV and the adsorption distance was 2.88 Å, as shown in Table 1. Moreover, the Mulliken charge analysis showed −0.104 e transfer between NO and ZGNR during the adsorption process. On the other hand, the adsorption energy, adsorption distance, and charge transfer values between NO_2_ and ZGNR were −0.225 eV, 3.11 Å, and 0.040 e, respectively. The positive value of charge transfer in the case of NO_2_ gas indicates that the electrons transfer from ZGNR to NO_2_. The results also reflect the capability of ZGNR to adsorb NH_3_ with −0.091 eV adsorption energy and −0.018 e charge transfer.

**Figure 2:**
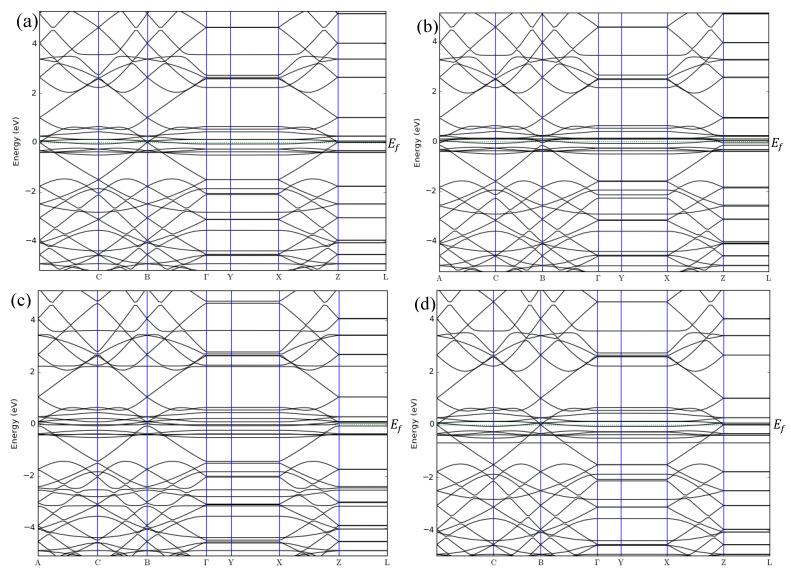
Band structures of (**a**) ZGNR, (**b**) ZGNR-NO, (**c**) ZGNR-NO_2_, and (**d**) ZGNR-NH_3_.

**Figure 3:**
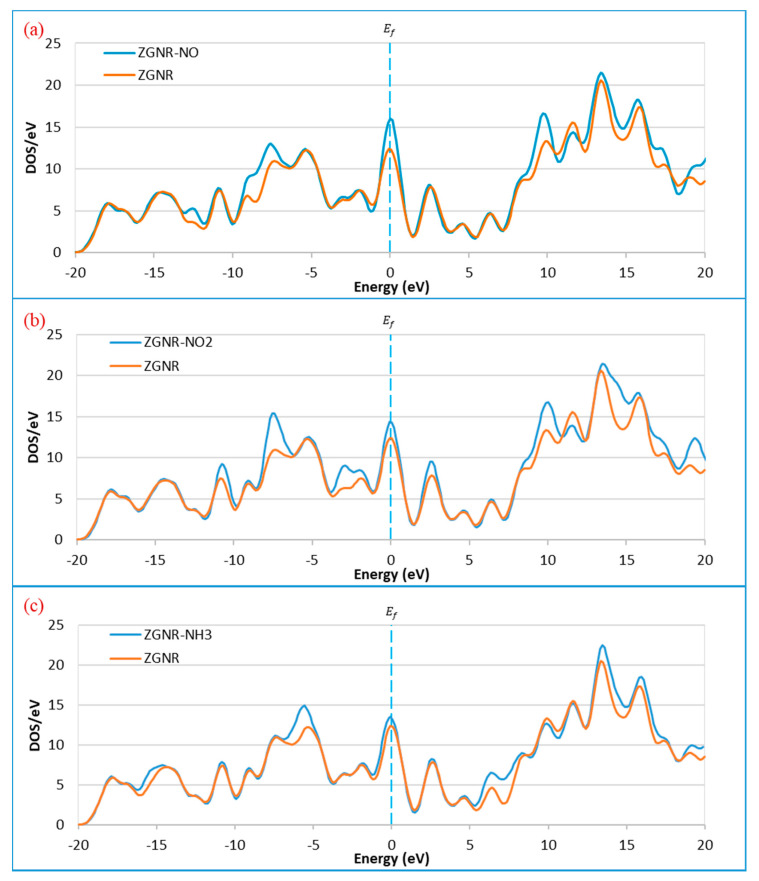
Density of states of the ZGNR system before and after the adsorption of (**a**) NO, (**b**) NO_2_, and (**c**) NH_3_.

The band structures of ZGNR before and after adsorption of NO, NO_2_, and NH_3_ gases are shown in Figure 2a–d. The band structure results show that the band gap of ZGNR is 0 eV, reflecting its metallic nature, which has been reported [51,52]. Although no changes in the band gap were observed after the adsorption of NO, NO_2_, and NH_3_ gases, considerable changes were detected in the DOS results in Figure 3. 

The DOS results show that the DOS at the Fermi level increases significantly after adsorption of NO and NO_2_ and slightly for the case of NH_3_. In addition to the increase of DOS at the Fermi level, a significant increase was also observed around −12.3, −7.4, 9.9, and 17.3 eV for the case of NO in Figure 3a. For the case of NO_2_ in Figure 3b, considerable increases of the DOS around −10.6, −7.4, −2.8, −1.9, 2.8, 9.9, and 19.2 eV were observed after gas adsorption. Meanwhile, increases of DOS for the case of NH_3_ were observed around −5.2, 6.5, 13.5, 15.8, and 19.2 eV, as shown in Figure 3c. 

### 3.2. ZGNR-O System

**Figure 4:**
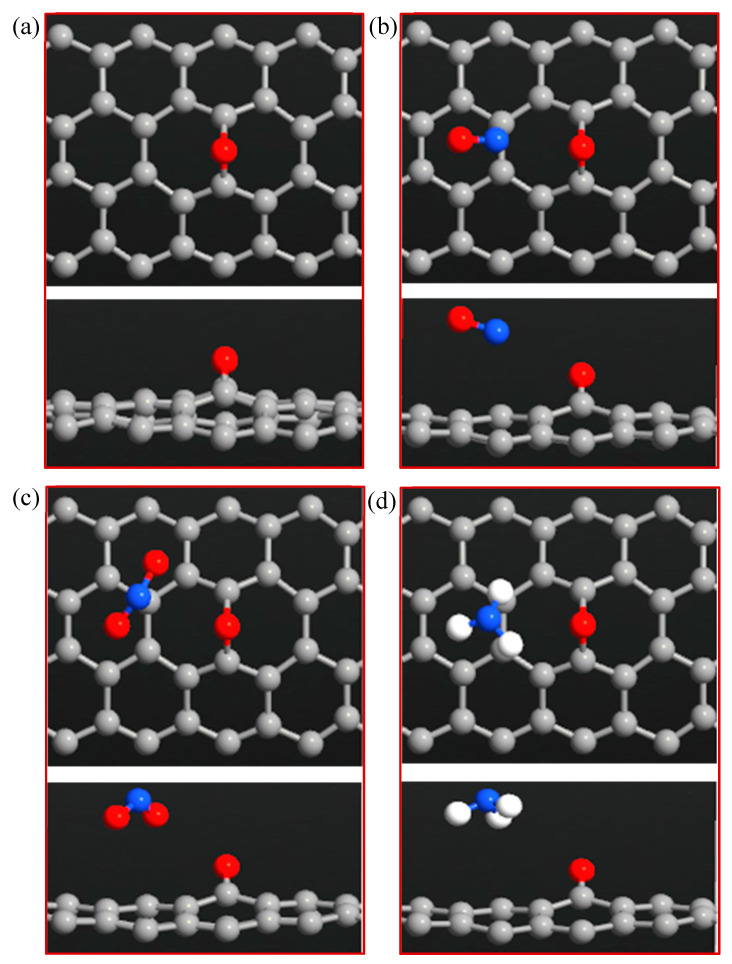
Top and side views of (**a**) ZGNR-O, (**b**) ZGNR-O-NO, (**c**) ZGNR-O-NO_2_, and (**d**) ZGNR-O-NH_3_.

**Figure 5:**
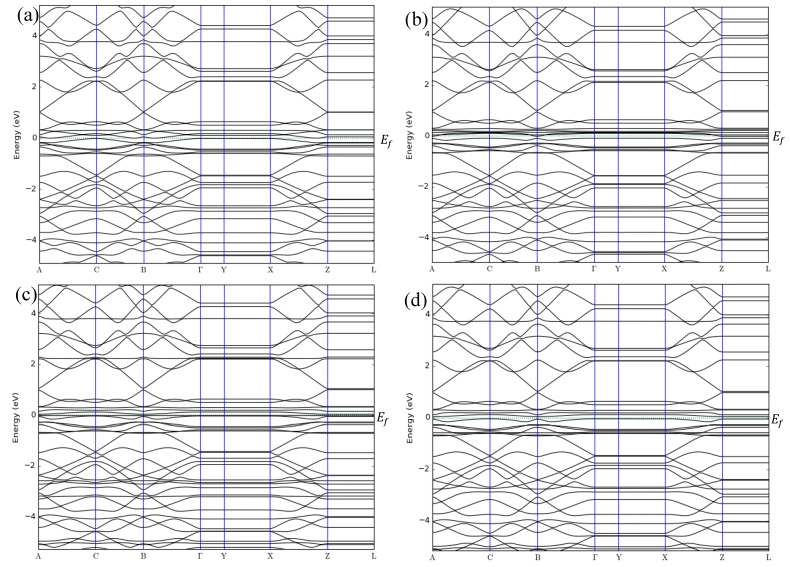
Band structures of (**a**) ZGNR-O, (**b**) ZGNR-O-NO, (**c**) ZGNR-O-NO_2_, and (**d**) ZGNR-O-NH_3_.

**Figure 6:**
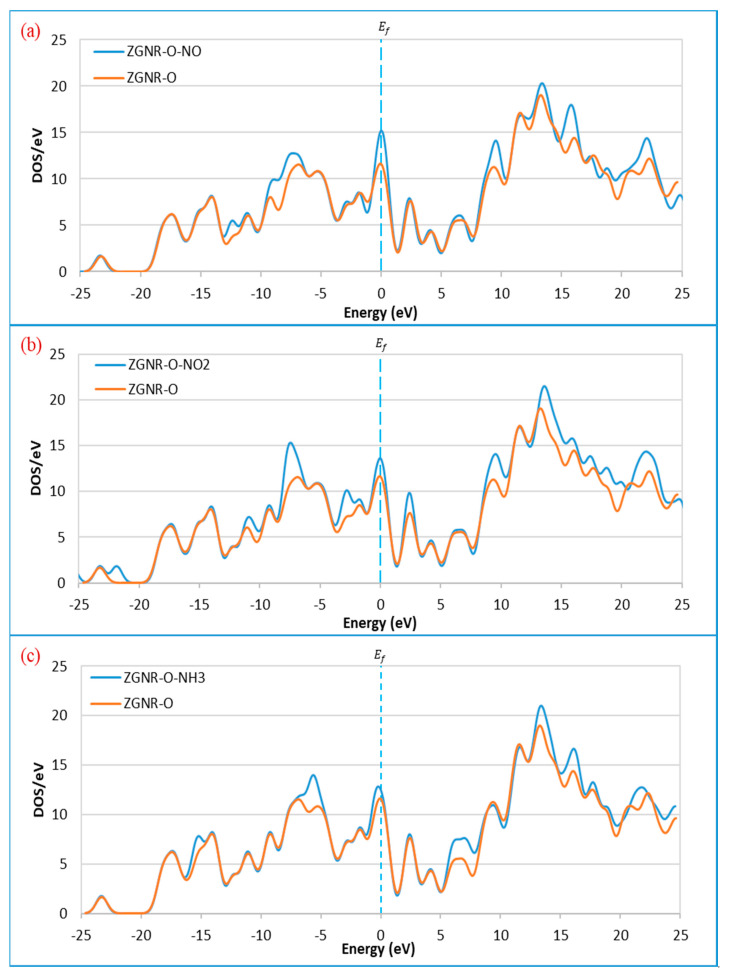
Density of states of the ZGNR-O system before and after the adsorption of (**a**) NO, (**b**) NO_2_, and (**c**) NH_3_.

Figure 4a–d shows the optimized structure of the ZGNR-O system before and after adsorption of the gas molecules. The results show that the C-O bond length was 1.39 Å. The adsorption energy, adsorption distance, and charge transfer between the gas molecules and ZGNR-O are shown in Table 2. The adsorption distances between NO, NO_2_, and NH_3_ gases and ZGNR-O were 2.66, 3.15, and 3.15 Å, respectively. Although no considerable changes were observed in the adsorption parameters after the adsorption of NO_2_ gas, after the functionalization of ZGNR with -O- group, the adsorption energy increased to −0.318 and −0.124 eV for the cases of NO and NH_3_. Moreover, the charge transfer between NH_3_ gas and ZGNR-O system increased significantly to −0.128 e. Figure 5a–d shows the band structures of ZGNR-O before and after adsorption of NO, NO_2_, and NH_3_ gases. No significant changes were observed in the band gap after the adsorption of the gas molecules in the band structure results. Nevertheless, a remarkable increase in the DOS at the Fermi level was observed after the adsorption of the three gases on the surface of ZGNR-O, as shown in Figure 6. Figure 6a shows that DOS values around −12.3, −9.0, −7.1, 9.6, 13.5, 16.0, and 22.3 eV increased significantly after adsorption of NO gas. Due to the adsorption of NO_2_, a new peak around −21.7 eV was detected, as shown in Figure 6b. In addition, considerable increases were observed around −10.9, −7.1, −2.6, 2.5, 9.6, 13.5, 16.0, 17.5, 18.8, and 22.3 eV, confirming the adsorption of NO_2_ gas. For the case of NH_3_ in Figure 6c, considerable increases in DOS values around −15.4, −5.2, 6.5, 13.5, 16.0, and 17.5 eV were observed. Moreover, the peaks around 20.8 and 22.4 eV were combined to form a new peak around 21.4 eV. 

### 3.3. ZGNR-OH System

**Figure 7:**
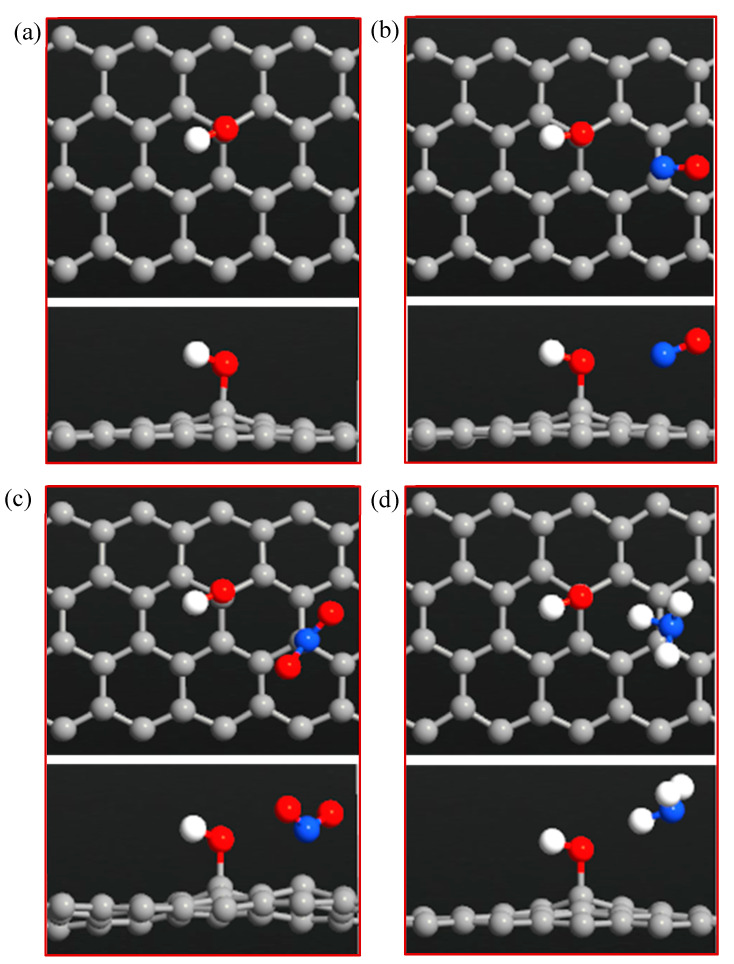
Top and side views of (**a**) ZGNR-OH, (**b**) ZGNR-OH-NO, (**c**) ZGNR-OH-NO_2_, and (**d**) ZGNR-OH-NH_3_.

In this part, the surface of ZGNR was functionalized with one -OH group and then used as a gas sensor to detect NO, NO_2_, and NH_3_ gases, as shown in Figure 7. The results show that the C-C bond length of ZGNR increased a little to 1.5 Å around the -OH group, while the C-O and O-H bonds were 1.49 Å and 0.98 Å, respectively. Table 3 shows the adsorption parameters of the optimized NO, NO_2_, and NH_3_ gases adsorbed on ZGNR-OH. On one hand, the adsorption distances between NO, NO_2_, and NH_3_ and ZGNR-OH decreased to 2.24, 1.74, and 2.18 Å, respectively. On the other hand, a significant improvement in the adsorption energy was observed with −0.641, −0.618, and −0.244 eV for the cases of NO, NO_2_, and NH_3_ gases, respectively, as compared with the ZGNR and ZGNR-O systems. Moreover, the Mulliken charge analysis showed that 0.146 and 0.137 e transfer from NO and NH_3_ to ZGNR-OH (negative ∆Q) during the adsorption process, while 0.074 e transfer from ZGNR-OH to NO_2_. Therefore, the sensing signal is expected to be negative for the former and positive of the latter.

**Figure 8:**
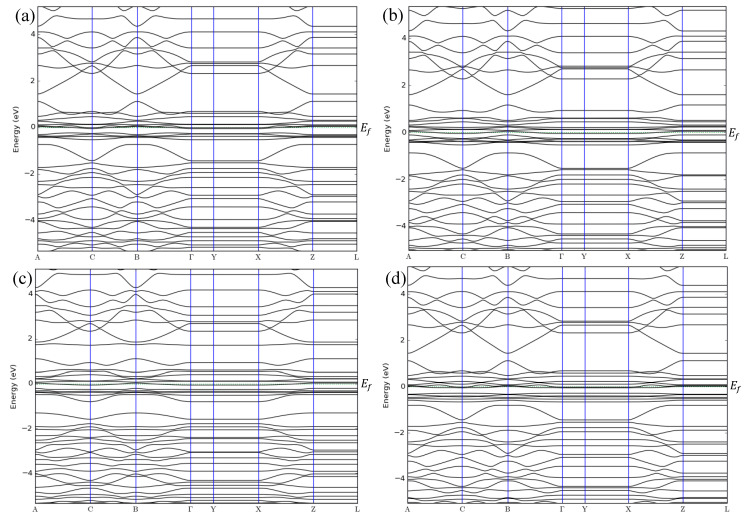
Band structures of (**a**) ZGNR-OH, (**b**) ZGNR-OH-NO, (**c**) ZGNR-OH-NO_2_, and (**d**) ZGNR-OH-NH_3_.

The band structures of ZGNR-OH before and after adsorption of NO, NO_2_, and NH_3_ gases are shown in Figure 8a–d, respectively. The results show no significant changes in the band gap after the adsorption of NO, NO_2_, and NH_3_ gases; however, some changes were detected below and above the Fermi level as a result of the gas adsorption. 

**Figure 9:**
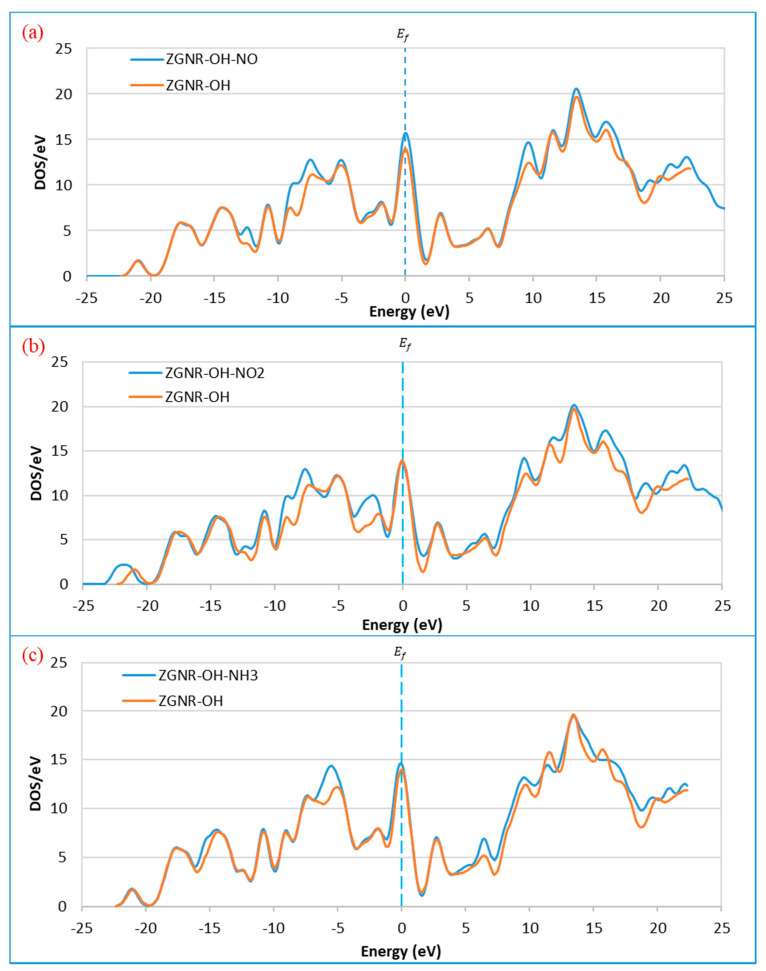
Density of states of the ZGNR-OH system before and after the adsorption of (**a**) NO, (**b**) NO_2_, and (**c**) NH_3_.

The DOS results in Figure 9 show that the DOS at the Fermi level increases after the adsorption of NO and NH_3_, reflecting an improvement of ZGNR-OH conductivity upon gas adsorption. The results reveal that the DOS around −12.2, −8.9, −7.4, and 9.8 eV increased after the adsorption of NO gas, as shown in Figure 9a, while two new peaks were observed around 21.1 and 22.2 eV. In addition, the peak around 19.9 eV shifted to 19.0 eV. Although no significant change was detected at the Fermi level after the adsorption on NO_2_ (Figure 9b), a considerable increase in the DOS around −8.9, −7.4, −2.1, 9.8, and 15.7 eV was detected. Moreover, the peak around −21.0 eV shifted to −21.8 eV, the peak around 19.9 eV shifted to 19.0 eV, and two new peaks around 21.1, and 22.2 eV were observed. Figure 9c shows that the DOS at Femi level increases slightly after the adsorption of NH_3_ gas. A significant increase in the DOS around −5.2 and 6.5 eV was also observed.

### 3.4. ZGNR-O-OH System

**Figure 10:**
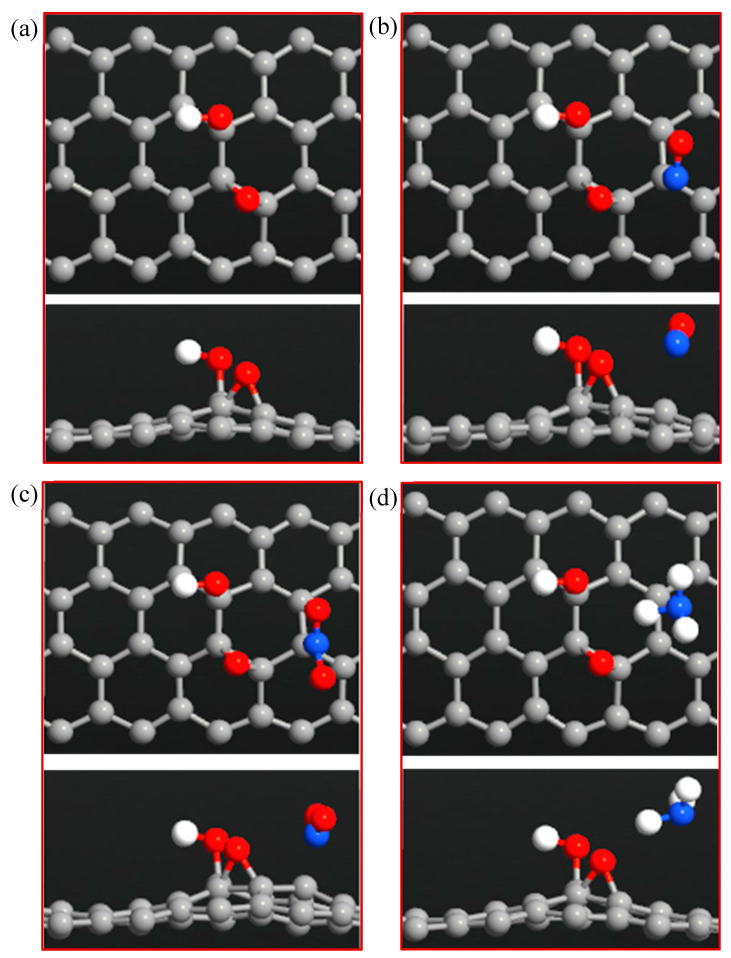
Top and side views of (**a**) ZGNR-O-OH, (**b**) ZGNR-O-OH-NO, (**c**) ZGNR-O-OH-NO_2_, and (**d**) ZGNR-O-OH-NH_3_.

To improve the adsorption capacity, the surface of ZGNR was functionalized with both -O- and -OH groups, as shown in Figure 10, and then used to detect NO, NO_2_, and NH_3_ gases. The adsorption energies, adsorption distances, and charge transfer of the optimized NO, NO_2_, and NH_3_ gases adsorbed on ZGNR-O-OH system are listed in Table 4. The results show that modifying the surface of ZGNR with -O- and -OH groups significantly enhanced its adsorption capacity. For instance, the adsorption distances decreased to 1.98, 1.68, and 2.45 Å, while the adsorption energies increased significantly to −0.625, −0.953, and −0.219 eV for the cases of the NO, NO_2_, and NH_3_ gases, respectively. On the other hand, the Mulliken charge analysis demonstrated that 0.118 and 0.141 e transfer from NO and NH_3_ to ZGNR-O-OH during the adsorption process, while 0.092 e transfer from ZGNR-O-OH to NO_2_ gas. 

**Figure 11:**
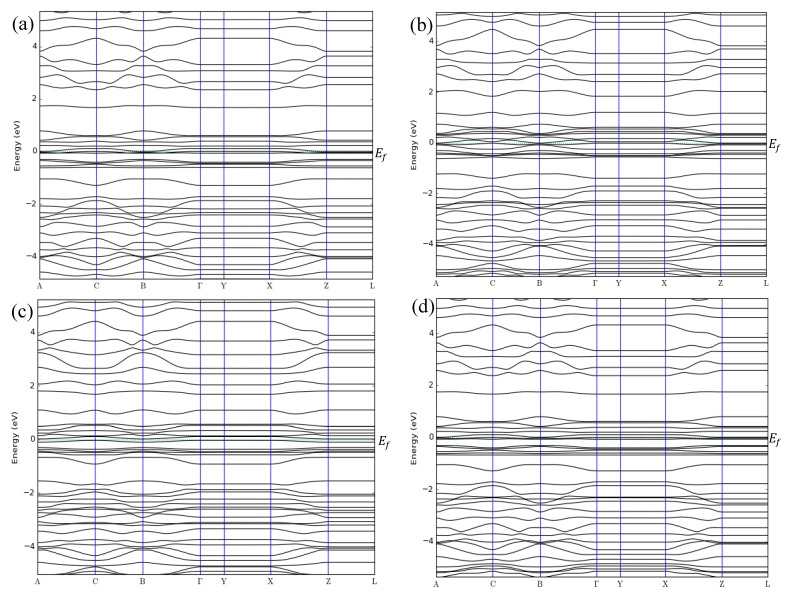
Band structures of (**a**) ZGNR-O-OH, (**b**) ZGNR-O-OH-NO, (**c**) ZGNR-O-OH-NO_2_, and (**d**) ZGNR-O-OH-NH_3_.

The band structures of the ZGNR-O-OH system before and after the adsorption of NO, NO_2_, and NH_3_ gases are shown in Figure 11a–d, respectively. The band structure results show that the band gap of ZGNR increased slightly to 0.043 eV after the functionalization with -O- and -OH groups. The band gap then decreased to 0 eV after the adsorption of the gas molecules, reflecting an improvement in its conductivity and the differentiation between the two cases before and after gas adsorption. Moreover, some other changes were observed below and above the Fermi level due to the adsorption of NO, NO_2_, and NH_3_ gases.

**Figure 12:**
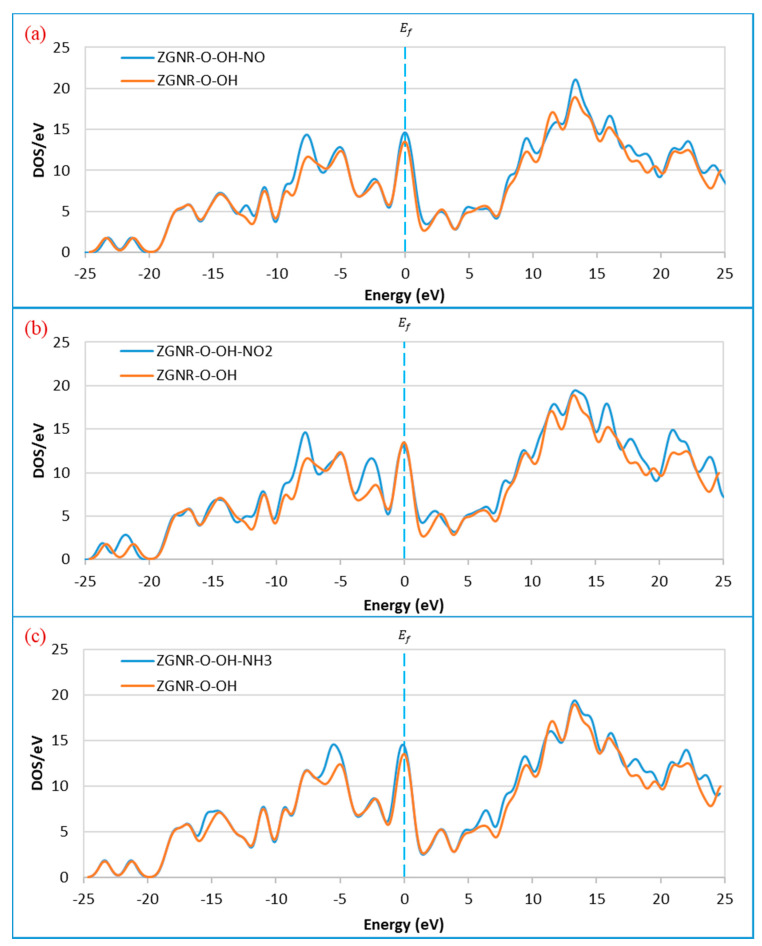
Density of states of the ZGNR-O-OH system before and after the adsorption of (**a**) NO, (**b**) NO_2_, and (**c**) NH_3_.

These changes are confirmed by the DOS results in Figure 12. The DOS results show that the DOS at Fermi level increased after the adsorption of NO and NH_3_. Figure 12a shows a remarkable increase in the DOS around −12.3, −7.5, 9.6, 13.5, 16.0, and 22.2 eV upon the adsorption of NO gas. The peaks around 18.3 and 19.7 eV shifted to around 17.6 and 18.9 eV and a new peak around 24.3 eV was observed, confirming the adsorption of NO gas on the surface of ZGNR-O-OH. For the case of NO_2_ gas in Figure 12b, a significant increase in the DOS around −7.5, −2.5, 16.0, 17.6, and 21.1 eV was detected. Moreover, the peak around −21.0 eV shifted to −21.8 eV and a new peak around 24.0 eV was observed. Figure 12c shows a significant increase in the DOS around −5.2, 6.5, 9.6, 18.2, 19.5, and 22.2 eV after the adsorption of NH_3_ gas. In addition, a new peak around 23.7 eV was also observed.

## 4. Discussion

To improve the adsorption capacity of ZGNR, which is goal in this study, the surface of ZGNR was functionalized with -O- and -OH groups, creating three different systems (ZGNR-O, ZGNR-OH, and ZGNR-O-OH). Interestingly, the adsorption parameters of ZGNR reflect a significant improvement upon surface functionalization. This improvement is attributed mainly to the presence of the functional groups, which have been reported to play a significant role in facilitating the adsorption of the gas molecules during the sensing process [53,54,55]. 

The Mullekin charge analysis showed that the charge transfer is negative for the cases of NO and NH_3_ gases, which is an indication that the charge transfers from the gas molecules to the ZGNR systems (negative sensing signal), which is in good agreement with the reported data stating that NO and NH_3_ gases behave as donors [5,56]. On the other hand, for the case of NO_2_ the charge transfer was positive, which means that the charge transfers from the ZGNR systems to the gas molecules (positive sensing signal), which have been reported to behave as an acceptor [5,56].

In addition to the adsorption energy, adsorption distance, and charge transfer, the adsorption of NO, NO_2_, and NH_3_ gases on the surface of the ZGNR systems was also confirmed by the band structure and density of states results. The band structure results show some changes in the bands before and after the Fermi level as a result of the gas adsorption. These changes confirm the successful adsorption of the gas molecules on the surface of ZGNR systems. Moreover, the DOS results demonstrated a significant increase of the density at Fermi level and the appearance of some new peaks. The significant increase in the density of states is related to the improvement of the ZGNR system conductivity upon gas adsorption, which is in good agreement with the reported data that relate the change of adsorbent’s conductivity after adsorption of gas molecules [57,58,59].

The sensing of nitrogen oxides can be assigned to their easy and rapid diffusion on the surface of ZGNR. This leads to formation of hydrogen bonds among the nitrogen oxides and ZGNR, which significantly contributes to increasing their binding energy. The interaction among nitrogen oxides with ZGNR with the epoxy group and hydroxyl groups can be attributed to the development of hydrogen bonds OH…O(N) between NO_x_ and -OH and the formation of new weak covalent bonds (C…N as well as C…O), as well as H elimination to produce nitrous acid- and nitric acid-like moieties [60,61].

## 5. Conclusions

The main goal of this study was to enhance the sensing performance of zigzag graphene nanoribbon (ZGNR) towards the detection of NO, NO_2_, and NH_3_ gases. First, primary calculations using density functional theory (DFT), based on Atomistic ToolKit Virtual NanoLab (ATK-VNL), were used to study the adsorption of the target gases on the surface of the ZGNR. The best sensing performance was found for the case of NO gas, with −0.273 eV adsorption energy and −0.104 e charge transfer during the adsorption process. In order to improve the sensing performance of ZGNR, three different functionalized systems were built: ZGNR-O, ZGNR-OH, and ZGNR-O-OH. The sensing performance towards the three gases was improved significantly after the functionalization, especially for the cases of the ZGNR-OH and ZGNR-O-OH systems. Nevertheless, the best sensing performance from the four systems based on the adsorption energy, adsorption distance, charge transfer, and conductivity improvement was identified for the case of NO gas. Among the four systems, ZGNR-OH with adsorption energy of −0.641 eV and −0.146 e charge transfer was the best. Thus, it is the most selective to NO gas, reflecting that ZGNR-OH can be considered as a promising gas sensor to detect NO gas.

## Figures and Tables

**Table 1:** Adsorption parameters of the optimized NO, NO_2_, and NH_3_ gases adsorbed on the zigzag graphene nanoribbon (ZGNR) system. $E_{ads}$: adsorption energy; D: adsorption distance; ∆Q: charge transfer.

Gas	$E_{ads}\;(\mathrm{eV})$	D (Å)	∆Q (e)
NO	−0.273	2.88	−0.104
NO_2_	−0.225	3.11	0.040
NH_3_	−0.092	3.03	−0.018

**Table 2:** Adsorption parameters of the optimized NO, NO_2_, and NH_3_ gases adsorbed on the ZGNR-O system.

Gas	$E_{ads}\;(\mathrm{eV})$	D (Å)	∆Q (e)
NO	−0.318	2.66	−0.132
NO_2_	−0.212	3.15	0.031
NH_3_	−0.124	3.15	−0.128

**Table 3:** Adsorption parameters of the optimized NO, NO_2_, and NH_3_ gases adsorbed on the ZGNR-OH system.

Gas	$E_{ads}\;(\mathrm{eV})$	D (Å)	∆Q (e)
NO	−0.641	2.24	−0.146
NO_2_	−0.618	1.74	0.074
NH_3_	−0.244	2.18	−0.137

**Table 4:** Adsorption parameters of the optimized NO, NO_2_, and NH_3_ gases adsorbed on the ZGNR-O-OH system.

Gas	$E_{ads}\;(\mathrm{eV})$	D (Å)	∆Q (e)
NO	−0.625	1.98	−0.118
NO_2_	−0.953	1.68	0.092
NH_3_	−0.219	2.45	−0.141
